# Cancer patients' awareness of clinical trials, perceptions on the benefit and willingness to participate: Korean perspectives

**DOI:** 10.1038/sj.bjc.6604750

**Published:** 2008-11-11

**Authors:** J W Kim, S-J Kim, Y-H Chung, J-H Kwon, H-J Lee, Y-J Chung, Y J Kim, Do-Youn Oh, S-H Lee, D-W Kim, S-A Im, T-Y Kim, D S Heo, Y-J Bang

**Affiliations:** 1 Department of Internal Medicine, Seoul National University Hospital, Seoul National University College of Medicine, Seoul, Korea; 2 Cancer Research Institute, Seoul National University College of Medicine, Seoul, Korea

**Keywords:** cancer patient, clinical trial, perception, awareness, willingness

## Abstract

To understand patients' perceptions of clinical trials (CTs) is the principal step in the enrolment of patients to CTs. However, these perceptions in eastern countries are very rare. From 12 February 2007 to 13 April 2007, we consecutively distributed the questionnaire to 842 cancer patients who initiated a first cycle of chemotherapy regardless of each treatment step in the Seoul National University Hospital. Younger age, higher educational degree, higher economic status, and possession of private cancer insurance were related with significantly higher awareness of CTs (*P*=0.001, *P*=0.006, *P*=0.002, and *P*=0.009, respectively). However, unlike awareness, perceptions on benefits of CTs were not changed according to age, educational degree, and economic status (*P*=0.709, *P*=0.920, and *P*=0.847, respectively). Willingness was also not changed according to age, educational degree, economic status, and private cancer insurance (*P*=0.381, *P*=0.775, *P*=0.887, and *P*=0.392, respectively). Instead, males and heavily treated patients had more positive perceptions on benefits (*P*=0.002 and *P*=0.001, respectively) and more willingness to participate in CTs (OR=1.17, 1.14–2.75: OR=1.59, 1.01–2.51, respectively). In summary, cancer patients' awareness of CTs, perceptions on the benefit in CTs, and willingness to participate are differently influenced by diverse medical and social conditions. This information would be very helpful for investigators to properly conduct CTs in eastern cancer patients.

Nowadays, the molecular biological characteristics of tumours have been thoroughly identified. On the basis of these results, many novel agents and modalities have been developed and are being studied for clinical use. Cancer clinical trials (CTs) are needed to establish new treatment options through evaluating the efficacy and toxicity of novel therapy. In addition, participation in CTs gives patients a chance to receive the most up-to-date therapy (Cobau, 1994). Participation in CTs is also frequently related to a higher survival rate (Stiller, 1994). Therefore, CTs are an important issue in oncology, and the need for such studies is continuously growing.

However, in the process of conducting CTs, there are some limitations, including administrative barriers, low accrual rate, ethical issues, problem with referral systems, low research funding, communication barriers, and other issues (Lara *et al*, 2001; Kaas *et al*, 2005; Dilts and Sandler, 2006; Ho *et al*, 2006). Of these limitations, low accrual rate is the most important barrier to conducting CTs. Sufficient numbers of patients to drive appropriate outcomes are a necessary component. However, CT participants represented approximately 1.7% of the total number of incident cancer cases during the study period from 2000 to 2002 in the United States (Murthy *et al*, 2004). In the East, accrual rate might also be low. Low accrual rates may also often prolong the duration of a CT, delay the analysis of an important outcome, or lead to early closure of CTs (Haidich and Ioannidis, 2001). Therefore, it is necessary to identify and overcome the barriers of the enrolment of patients into CTs.

To increase patient participation in CTs, it is very important to understand the perceptions of cancer patients regarding CTs. A few studies regarding accrual barriers of CTs reported that participation in CTs was influenced by social, economic, and cultural backgrounds as well as medical decisions (Lara *et al*, 2001, 2005; Sateren *et al*, 2002; Paterniti *et al*, 2005; Tu *et al*, 2005). Most studies addressing the perceptions of cancer patients on CTs have been conducted in western countries (Comis *et al*, 2003) and study populations were usually limited to particular cancer types or participants in phase I research (Weeks *et al*, 1998; Meropol *et al*, 2003; Agrawal *et al*, 2006). Considering the social and cultural differences between eastern and western countries, it is difficult to directly extrapolate outcomes obtained in the West to the people of the East. Moreover, the number of CTs conducted in the East has recently begun to increase rapidly. However, the prospective data addressing these issues in eastern populations remain quite limited.

Thus, we conducted this prospective study to determine how cancer patients view CTs according to patient conditions and how these perceptions influence the willingness of cancer patients to participate in CTs in the East.

## Patients and methods

### Patients

From 12 February 2007 to 13 April 2007, we prospectively surveyed cancer patients who initiated the first cycle of their chemotherapy at Seoul National University Hospital in Korea. For example, patients who started their adjuvant chemotherapy, first-line palliative chemotherapy, second-line palliative chemotherapy, and so on were consecutively enrolled.

With the agreement of each patient, personal information was gathered. Disease status was retrieved from medical records. These perceptions from patients were compared with those of physicians. We received informed consent from all individuals before enrolment in this study.

### Questionnaire

A questionnaire was distributed to cancer patients to be matched with the study criteria consecutively. For patients to be enrolled in actual cancer CT, their physicians also received the questionnaire to assess their perceptions on CTs. The contents of the questionnaire included: (1) patients' awareness of cancer CTs and sources of information for cancer CTs, (2) perceptions of patients and physicians associated with the benefits of CTs compared with conventional therapy, and (3) willingness of patients to participate in CTs and the reason of participation or non-participation in cancer CT.

### Quantitative benefit of participation in cancer CT

A visual analogue scale (VAS) between 0 and 10 was used in a survey of the perceptions on the effect of each treatment. In the VAS score, zero points indicate ‘never effective’ and 10 points indicate ‘effective enough to cure’. The ‘benefits’ of participating in CTs were calculated with the difference of the VAS score between a CT and a conventional therapy (benefit=effect of CT–effect of conventional therapy).

### Statistical analysis

The *χ*^2^-test was used in the analysis of awareness and willingness according to patient characteristics. To show perceptions on the benefit of a CT, the VAS score with multiple factors was analysed by *t*-test or one-way analysis of variance (ANOVA). Odds ratios obtained by logistic regression revealed the influence of patients' awareness and the benefit of the CT on the willingness to participate. Multivariate analysis was also conducted by logistic regression. To evaluate the difference of perceptions between patients and physicians regarding the effect of treatment, VAS scores of both groups were analysed by paired *t*-test. All analyses were performed using SPSS for Windows, version 12.0 (SPSS Inc., Chicago, IL, USA).

## Results

A total of 842 cancer patients were enrolled who initiated their first cycle of chemotherapy. Forty-seven CTs were available for enrolment during this study period in Seoul National University Hospital. In all, 12.4% of total patients participated in cancer CTs during study period (105 out of 842). Among 842 patients, 524 patients completed the questionnaire (62.1%). Among 105 patients who were actually enrolled in cancer CTs, 42.9% responded to the questionnaire (45 out of 105). In contrast, among patients who were not enrolled in cancer CTs, the rate of responders to the questionnaire was higher than in real participants in cancer CTs (479 out of 737, 65.0%; *P*<0.001).

### Patients' characteristics of responders for questionnaire

Males constituted 45.6% of the population. Among patients, 30.5 and 32.3% were in a first-line setting of palliative chemotherapy and adjuvant chemotherapy setting, respectively. Breast cancer was the most common cancer type (168, 32.1%), and most patients ranged in age from 40 to 70 years (410, 78.2%). In terms of the degree of education, 71.6% of the population had gone beyond high school. Most patients were married and lived within a 2-h distance of the hospital (81.1 and 66.4%, respectively). The rate of patients with private cancer insurance was 48.3% (253). The economic status of patients was evenly distributed (Table 1).

### Cancer patients' awareness of CTs

For the question ‘Have you ever heard about cancer CTs?’, 433 (82.6%) individuals answered ‘yes’. Mass media such as TV, internet, and newspapers was the most important source of information (68%). The second source was a physician (22%). The others were relatives and other patients (15 and 6%, respectively). Younger age, higher educational degree, higher economic status, and possession of private insurance were associated with a higher rate of awareness for CTs (*P*=0.001, *P*=0.006, *P*=0.002, and *P*=0.009, respectively). However, disease status and marital status were not related to awareness of CTs (Table 2).

### Perceptions of cancer patients regarding the effects of CTs compared with conventional therapy

**Figure 1:**
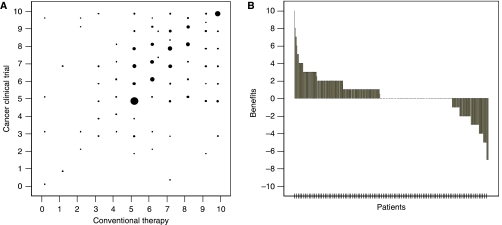
Visual analogue scale (VAS) on effects of clinical trial (CT) and conventional therapy. (**A**) Distribution. The size of solid circle indicates the number of patients. (**B**) The benefit of participation in clinical trials. A positive value indicates a higher expectation of benefits for the clinical trial therapy. Zero points indicate equal expectation of both therapies.

In the evaluation of the perceptions on effects of treatments using a VAS, most patients marked over five points (Figure 1). Figure 1 also revealed that most patients had positive perceptions on the effects of CTs as compared with conventional therapy (mean VAS=6.95±2.00 and 6.56±2.04, respectively; *P*<0.001, benefit=0.38±2.09). Both males and females had positive perceptions but males had more positive perceptions than females (benefit=0.74±2.07 and 0.11±2.07, respectively; *P*=0.002). In the group of more heavily treated patients, patients favoured the treatments offered in CTs more positively (*P*=0.001). However, in terms of neoadjuvant and adjuvant disease status, patients had negative views of the treatments used in CTs (benefit=−0.12±1.71 and −0.10±2.36, respectively). Patients without private cancer insurance had more positive perceptions on the effects of CTs than patients with private cancer insurance (benefit=0.58±1.90 and 0.16±2.25, respectively; *P*=0.043). Other variables such as age, educational degree, economic status, and type of religion did not influence the perceptions of patients regarding the benefits of CTs as compared with conventional therapy (Table 3). Awareness of CTs did not also affect the perceptions of patients regarding the benefits of CTs (benefit=0.40±2.12 for patients with awareness of CTs and 0.26±1.95 for patients without awareness; *P*=0.631).

### Willingness of cancer patients to participate in CTs

If CTs were available and a recommendation was given from their physician, 64.7% patients answered that they would be willing to participate in CTs. These patients mostly believed that participation in CTs was the best treatment choice and involved state-of-the-art treatments (46%). Some patients answered that they would not decline participation just to have no other treatment options, or that they wanted to participate because of recommendations from their relatives (20 and 20%, respectively). A few patients reported the reasons to be the economic benefit of participation (14%). In contrast, the patients who answered that they did not want to participate in CTs believed that therapies used in CTs were not yet proven to be more effective than conventional therapies (54%). Some patients answered that they would not want to be experimental tools for new treatments (27%). Others were concerned about adverse effects of the treatment used in CTs (11%). Fear of randomisation was mentioned by 7% of individuals (14). A few patients refused to participate because of the opinions of family members (4%).

Males were more likely to participate in CTs than females (*P*=0.023). In adjuvant, neoadjuvant disease status, and first-line palliation, the rates of willingness to participate in CTs were lower than that of heavily treated metastatic cancer patients (*P*=0.003; Table 4). In multivariate analysis, male and heavily treated patients were also more willing to participate in cancer CTs (OR=1.17, 1.14–2.75, *P*=0.01: OR=1.59, 1.01–2.51, *P*=0.04, respectively; Table 5). Other factors, including economic status, educational degree, marital status, distance from home to hospital, and possession of private cancer insurance did not influence the willingness to participate in clinical trials. Overall, patients' awareness of clinical trials influenced the willingness to participate in clinical trials (OR=1.938, 95% CI: 1.22–3.07, *P*=0.005). However, in contrast to the increased awareness in younger patients, those with a higher educational degree, higher economic status, and possession of private cancer insurance, the willingness was not increased in these conditions (*P*=0.381, *P*=0.775, *P*=0.887, and *P*=0.392, respectively). In terms of benefits and willingness, the benefit of participation in CTs as compared with conventional therapy also influenced the willingness to participate in CTs (OR=1.31, 95% CI: 1.174–1.463, *P*<0.001). The more positive perception of benefits in males and heavily treated patients was in line with the findings of the willingness of patients to participate in CTs.

### Differences in the perception on the effects of treatment between cancer patients and their physicians

The physicians also answered questions about how much their patients might benefit from each therapy. Forty-five paired answers from both patients and their physicians were collected for perceptions on the effects of conventional therapy, and 42 paired answers were received for perceptions of CTs. For each of the conventional therapies and treatments used in CTs, physicians had less positive perceptions than the patients did (*P*<0.01 and *P*=0.028, respectively; Table 6). However, despite a lack of significance, physicians thought that participation in CTs would be more beneficial over conventional therapy than patients thought (benefit=1.14±1.60 and 0.76±2.49, respectively; *P*=0.229).

## Discussion

To our knowledge, this study represents the first large prospective study for perceptions of CTs in eastern countries. Considering that the number of cancer CTs and the proportion of global trials performed in the East are increasing rapidly, this subject should be interesting in the East. To evaluate sound perceptions of cancer patients at the step of actual enrolment, eligible patients were limited only to those at the start of the first cycle of their chemotherapy, who were usually considered to be actual candidates for participation in CTs. The most earlier studies regarding cancer patients' perceptions of CTs were reported in particular cancer types, patients in phase I trials, or the general population (Weeks *et al*, 1998; Comis *et al*, 2003; Meropol *et al*, 2003; Agrawal *et al*, 2006). Patients' perceptions could easily be changed according to patient conditions when faced with the recommendation to participate in CT (Gotay, 1991; Ross *et al*, 1999; Ellis, 2000). Therefore, it is valuable to confine this study to patients who could actually be considered as candidates of CTs at the point of enrolment. In addition, the perceptions regarding the effects of CTs and conventional therapy were quantified using VAS. Then, the benefit of participation in CTs was calculated through VAS scores. This quantitative approach was unique and decisive to define the difference of perceptions in various conditions.

In general, cancer CTs may be a matter of common interest. In this study, the mass media strongly influenced the awareness of CTs. Therefore, campaigning through mass media could be a good tool to increase the awareness of CTs. In fact, efforts have been made to increase the awareness of cancer CTs through internet websites in the United States. The 22% of sources from physicians might indirectly reflect the proportion of physicians' recommendations for their patients to participate in CTs. It was recently reported that physicians were the main resource of patients' awareness and, in addition, strongly influenced participation in cancer CTs (Comis *et al*, 2006).

Overall, willingness appeared to significantly reflect the awareness and perceptions on the benefit of CTs in this study. These results were in concordance with the relationship between awareness and willingness shown in earlier reports (Comis *et al*, 2003; Lara *et al*, 2005). However, awareness did not reflect perceptions on the benefit. From this finding, it could be inferred that patients' awareness does not necessarily indicate positive perceptions of CTs.

Participation in cancer CTs has increased overall since the early 1990s, but this has not been the case in cancer populations in low socioeconomic groups, the elderly, and ethnic minorities (Kaluzny *et al*, 1993; Trimble *et al*, 1994; Tejeda *et al*, 1996; Sateren *et al*, 2002; Christian and Trimble, 2003; Murthy *et al*, 2004). However, in this study, the rate of willingness to participate in CTs and the positive perceptions on the benefits of participation were not lower in the elderly or in patients with a lower socioeconomic status group, despite the fact that they had lower rates of awareness of CTs. Therefore, a lower accrual rate for CTs might originate from fewer opportunities to participate in CTs offered by investigators in the elderly or in the poor socioeconomic group, rather than the reluctance of patients to participate in CTs.

Heavily treated patients had more optimistic views of CTs and were more willing to participate in CTs compared with less heavily treated patients. These findings are supportive of an earlier study, in which patients facing life-threatening illnesses would weigh potential benefits and discount the risks associated with treatment (Gaskin *et al*, 1998). From these results, it could be expected that, in CTs with heavily treated cancer patients, it was not relatively difficult to enrol patients. In addition, because of negative views of cancer CTs in adjuvant, neoadjuvant, or early treatment steps, more comprehensive explanation of the benefits and aims of CT has to be given to candidates before they were willing to be enrolled in CT.

Insurance and funding also influenced the accrual of patients in cancer CTs (Lara *et al*, 2001, 2005). Possession of private cancer insurance cannot only reflect the concern for economic problems, but may also reflect health care issues. Therefore, it was explained that patients with private cancer insurance had greater awareness of CTs in this study. However, this higher awareness in patients with private cancer insurance was not linked with willingness to participate in CTs. These patients with private cancer insurance might be most likely to doubt the benefits of CTs, even though CTs tended to offer lots of benefits.

Another study mentioned the distance from the clinic as one of the reasons for accrual barriers (Lara *et al*, 2001). Although this was not significant, this tendency was shown in this study. Therefore, effective measures to offer a chance for patients far from a clinic to participate in CTs should be sought.

In terms of reasons of participation in CT, some agreed to participate because of recommendation by relatives (20%). A few patients refused to participate because of the opinions of family members (4%). These might reflect the culture in Korea, that is, lower patient autonomy in the treatment decision-making process. Most patients who did not want to participate in CTs were concerned about the efficacy and effectiveness of CTs. However, the aim of CT is usually the verification of the efficacy of a new treatment modality based on preclinical study and logical rationale that the CT therapy could be superior to or at least not inferior to conventional therapy. Therefore, these perceptions of cancer patients could be changed with comprehensive explanations of CTs.

In concordance with another earlier reports (Weeks *et al*, 1998; Meropol *et al*, 2003), patients had a more optimistic view for the effect of each of the treatments than physicians expected. These results indicate the possibility of potential communication barriers between patients and physicians. However, physician thought that participation in cancer CTs was more beneficial as compared with patients thought. Physicians should carefully consider the expectancy of patients in CTs to manage the compliance of patients in CTs.

The limitation of this study is that the level of awareness was not evaluated. In an earlier report, the level of knowledge was significantly associated with willingness to participate in CTs (Lara *et al*, 2005). It could be completely expected that the quality and quantity of awareness directly influenced the willingness of patients to participate in CTs from this study. Another limitation is selection bias. Our data did not include all the patients of candidates for study period and only 62% patients of all the candidates in this study responded to the questionnaire. This low response rates might be resulted from the perplexed condition at that time of request for questionnaire because patients were noticed that their disease progressed or that new cancer diagnosed. In these bewildered situations, patients were uncooperative to respond to questionnaire. This study has been conducted in one hospital of Korea. Therefore, it could not be easy that the data of this study include all eastern perceptions of CT. Nevertheless, these data could give evidence that is representative of the perceptions in eastern countries.

This study addresses that perceptions of cancer patients such as awareness, perception on the benefit of CTs, and willingness to participate in CTs are influenced by socioeconomic background, disease status, age, and sex. These results would be conducive to physicians' understanding proper patients' perceptions of CTs in pertinent conduct of cancer CTs in the East.

## Figures and Tables

**Table 1:** Baseline characteristics

**Variable**	**Frequency (*N*=524)**	**%**
*Sex*		
Male	239	45.6
Female	285	54.4
		
*Age (years)*		
⩽30	12	2.3
30< to ⩽40	59	11.3
40< to ⩽50	121	23.1
50< to ⩽60	128	24.4
60< to ⩽70	161	30.7
70<	43	8.3
		
*Disease status*		
Neoadjuvant	25	4.8
Adjuvant	169	32.3
Metastatic, first line	160	30.5
Metastatic, second line	79	15.1
Metastatic, third line	49	9.4
Metastatic, fourth line	21	4.0
Metastatic, fifth line	11	2.1
Metastatic, sixth line	8	1.5
Metastatic, seventh line	2	0.4
		
*Educational degree*		
Postgraduate	37	7.1
University	153	29.2
High school	185	35.3
Middle school	57	10.9
Elementary school	58	11.1
Uneducated	10	1.9
Unknown	24	4.6
		
*Religion*		
Buddhism	162	30.9
Christian	153	29.2
Catholicism	74	14.1
Others	79	15.1
Unknown	56	10.7
		
*Diagnosis*		
Breast	168	32.1
Lung	98	18.7
Stomach	47	9.0
Colorectal	70	13.4
Hepatocellular	14	2.7
Biliary	21	4.0
Pancreas	14	2.7
Lymphoma	12	2.3
Head and neck	23	4.4
Sarcoma	11	2.1
Gynecology and genitourinary	11	2.1
Oesophagus	8	1.5
Others^a^	27	5.2
		
*Marital status*		
Single	34	6.5
Married	425	81.1
Bereavement	29	5.5
Divorced	20	3.8
Unknown	16	3.1
		
*Economic status*		
>5^b^	64	12.2
5⩾ to >4	46	8.8
4⩾ to >3	78	14.9
3⩾ to >2	58	11.1
2⩾ to >1	93	17.7
1⩾	129	24.6
Unknown	56	10.7
		
*Distance from clinic*		
⩽2 h	348	66.4
>2 h	176	33.6
		
*Private cancer insurance*		
Yes	253	48.3
No	248	47.3
Unknown	23	4.4

a Thyroid cancer, thymic carcinoma, germ cell tumour, skin cancer, neuroendocrine carcinoma, mesothelioma, anal cancer, brain, melanoma, renal cell carcinoma, metastatic of unknown origin, and small bowel cancer.

b Thousands United States dollars per month.

**Table 2:** Patients' awareness of cancer clinical trials

**Variable**	**Awareness of clinical trial (%)**	***P*-value**
*Sex*		
Male	194/239 (81.2)	0.418^a^
Female	239/285 (83.9)	
		
*Age (years)*		
⩽30	11/12 (91.7)	0.001^b^
30< to ⩽40	54/59 (91.5)	
40< to ⩽50	103/121 (85.1)	
50< to ⩽60	112/128 (87.5)	
60< to ⩽70	121/161 (75.2)	
70<	32/43 (74.4)	
		
*Disease status*		
Neoadjuvant	19/25 (76.0)	0.186^b^
Adjuvant	134/169 (79.3)	
Metastatic, first line	129/161 (80.1)	
Metastatic, second line	72/78 (92.3)	
Metastatic, third line	40/49 (81.6)	
Metastatic, fourth line	19/21 (90.5)	
Metastatic, fifth line	10/11 (90.9)	
Metastatic, sixth line	8/8 (100.0)	
Metastatic, seventh line	2/2 (100.0)	
		
*Marital status*		
Single	30/34 (88.2)	0.144^a^
Married	354/425 (83.3)	
Bereavement	20/29 (69.0)	
Divorced	15/20 (75.0)	
		
*Educational degree*		
Postgraduate	33/37 (89.2)	0.006^b^
University	131/153 (85.6)	
High school	153/185 (82.7)	
Middle school	49/57 (86.0)	
Elementary school	40/58 (69.0)	
Uneducated	7/10 (70.0)	
		
*Economic status*		
>5^c^	56/64 (87.5)	0.002^b^
5⩾ to >4	42/46 (91.3)	
4⩾ to >3	70/78 (89.7)	
3⩾ to >2	51/58 (87.9)	
2⩾ to >1	75/93 (80.6)	
1⩾	97/129 (75.2)	
		
*Religion*		
Buddhism	128/167 (79.0)	0.018^a^
Christian	135/153 (88.2)	
Catholicism	64/74 (86.5)	
Others	58/79 (73.4)	
		
*Private cancer insurance*		
Yes	221/253 (87.4)	0.009^a^
No	195/248 (78.6)	
		
*Distance from clinic*		
⩽2 h	286/348 (82.2)	0.702^a^
>2 h	147/176 (83.5)	

a Pearson *χ*^2^.

b Linear-by-linear association.

c Thousands United States dollars per month.

**Table 3:** Benefits of the effect of clinical trial therapy as compared with conventional treatment

**Variable (*N*)**	**Benefit (mean±s.d.)**	***P*-value**
*Sex*		
Male (184)	0.74±2.07	0.002^a^
Female (244)	0.11±2.07	
		
*Age (years)*		
⩽30 (11)	1.18±1.47	0.709^b^
30< to ⩽40 (51)	0.41±2.09	
40< to ⩽50 (108)	0.19±2.15	
50< to ⩽60 (106)	0.31±2.32	
60< to ⩽70 (118)	0.43±1.78	
70< to (34)	0.73±2.28	
		
*Line*		
Neoadjuvant (20)	−0.12±1.71	0.001^b^
Adjuvant (151)	−0.10±2.36	
Metastatic, first line (121)	0.47±1.62	
Metastatic, second line (58)	0.58±1.77	
Metastatic, third line (38)	0.77±2.21	
Metastatic, fourth line (20)	1.25±1.65	
Metastatic, fifth line (10)	1.70±2.35	
Metastatic, sixth line (8)	2.00±3.33	
Metastatic, seventh line (2)	2.00±2.82	
		
*Marital status*		
Single (28)	1.14±1.26	0.028^b^
Married (353)	0.38±2.11	
Bereavement (23)	−0.47±2.23	
Divorced (15)	1.00±1.13	
		
*Educational degree*		
Postgraduate (34)	0.32±1.42	0.92^b^
University (135)	0.32±2.24	
High school (152)	0.53±1.96	
Middle school (44)	0.31±2.34	
Elementary school (41)	0.31±1.91	
Uneducated (7)	−0.14±2.54	
		
*Economic status*		
>5^c^ (55)	0.36±1.97	0.847^b^
5⩾ to >4 (40)	0.07±2.11	
4⩾ to >3 (68)	0.33±2.46	
3⩾ to >2 (51)	0.52±1.75	
2⩾ to >1 (79)	0.60±1.73	
1⩾ (104)	0.41±2.25	
		
*Religion*		
Buddhism (130)	0.37±2.29	0.717^b^
Christian (129)	0.22±2.03	
Catholicism (64)	0.59±2.02	
Others (65)	0.40±2.04	
		
*Private cancer insurance*		
Yes (207)	0.16±2.25	0.043^a^
No (203)	0.58±1.90	
		
*Distance from Clinic*		
⩽2 h (288)	0.49±1.87	0.129^a^
>2 h (140)	0.16±2.46	
		
*Awareness of clinical trial*		
Yes (366)	0.40±2.12	0.631^a^
No (62)	0.26±1.95	

a *t*-test.

b One-way analysis of variances.

c Thousands United States dollars per month.

**Table 4:** Willingness of patients to participate in cancer clinical trials

**Variable**	**Willingness for clinical trial (%)**	***P*-value**
*Sex*		
Male	163/233 (70.0)	0.023^a^
Female	167/277(60.3)	
		
*Age (years)*		
⩽30	8/12 (66.7)	0.381^b^
30< to ⩽40	36/59 (61.0)	
40< to ⩽50	68/114 (59.6)	
50< to ⩽60	87/125 (69.6)	
60< to ⩽70	103/157 (65.6)	
<70	28/43 (65.1)	
		
*Line*		
Neoadjuvant	14/25 (56.0)	0.003^b^
Adjuvant	93/167 (55.7)	
Metastatic, first line	105/156 (67.3)	
Metastatic, second line	54/76 (71.1)	
Metastatic, third line	35/46 (76.1)	
Metastatic, fourth line	14/20 (70.0)	
Metastatic, fifth line	8/10 (80.0)	
Metastatic, sixth line	6/8 (75.0)	
Metastatic, seventh line	1/2 (50.0)	
		
*Marital status*		
Single	22/34 (64.7)	0.886^a^
Married	268/415 (64.6)	
Bereavement	20/29 (69.0)	
Divorced	13/18 (72.2)	
		
*Educational degree*		
Postgraduate	24/36 (66.7)	0.775^b^
University	95/152 (62.5)	
High school	119/179 (66.5)	
Middle school	35/53 (66.0)	
Elementary school	35/57 (61.4)	
Uneducated	8/10 (80.0)	
		
*Economic status*		
>5^c^	38/61 (62.3)	0.887^b^
5⩾ to >4	31/46 (67.4)	
4⩾ to >3	50/75 (66.7)	
3⩾ to >2	39/58 (67.2)	
2⩾ to >1	56/90 (62.2)	
1⩾	83/125 (66.4)	
		
*Religion*		
Buddhism	94/159 (59.1)	0.205^a^
Christian	92/146 (63.0)	
Catholicism	53/73 (72.6)	
Others	53/78 (67.9)	
		
*Private cancer insurance*		
Yes	152/243 (62.6)	0.392^a^
No	163/246 (66.3)	
		
*Distance from clinic*		
⩽2 h	226/337 (67.1)	0.120^a^
>2 h	104/173 (60.1)	
		
*Awareness of clinical trial*		
Yes	284/421 (67.5)	0.004^a^
No	46/89 (51.7)	

a Pearson *χ*^2^.

b Linear-by-linear association.

c Thousands United States dollars per month.

**Table 5:** Multivariate analysis for willingness

**Variable**	***P*-value**	**Odds ratio**	**95% CI**
Male	0.01	1.17	1.14–2.75
Old age (⩾60)	0.36	0.80	0.50–1.28
Heavily treated^a^	0.04	1.59	1.01–2.51
High educational degree (⩾university)	0.25	0.77	0.49–1.20
High economic status (⩾3 thousands United States dollars per month)	0.49	1.16	0.75–1.81
Private cancer insurance	0.60	0.89	0.58–1.37
>2 h, distance from clinic	0.06	0.67	0.44–1.02

a ⩾ second-line palliative therapy (*vs* adjuvant, neoadjuvant, and first-line palliative chemotherapy).

**Table 6:** Different view between patients and physicians regarding effect of treatment

**Variable (*N*)**	**Patients (mean±s.d.)**	**Physicians (mean±s.d.)**	***P*-value**
Conventional therapy (45)	6.37±2.04	5.08±2.02	<0.01^a^
Cancer clinical therapy (42)	7.14±1.84	6.28±1.83	0.028^a^
Benefit (42)	0.76±2.49	1.14±1.60	0.229^a^

a Paired *t*-test.
